# Long-term trends in the body condition of parents and offspring of Tengmalm’s owls under fluctuating food conditions and climate change

**DOI:** 10.1038/s41598-021-98447-1

**Published:** 2021-09-23

**Authors:** Marek Kouba, Luděk Bartoš, Jitka Bartošová, Kari Hongisto, Erkki Korpimäki

**Affiliations:** 1 Section of Ecology, Department of Biology, University of Turku, Turku, Finland; 2 Department of Ethology and Companion Animal Science, Faculty of Agrobiology, Food and Natural Resources, Czech University of Life Sciences Prague, Prague, Czech Republic; 3 Department of Ethology, Institute of Animal Science, Prague, Czech Republic; 4 Independent Researcher, Tampere, Finland

**Keywords:** Animal behaviour, Behavioural ecology, Boreal ecology

## Abstract

Physical condition is important for the ability to resist various parasites and diseases as well as in escaping predators thus contributing to reproductive success, over-winter survival and possible declines in wildlife populations. However, in-depth research on trends in body condition is rare because decades-long datasets are not available for a majority of species. We analysed the long-term dataset of offspring covering 34 years, male parents (40 years) and female parents (42 years) to find out whether the decline of Tengmalm’s owl population in western Finland is attributable to either decreased adult and/or juvenile body condition in interaction with changing weather conditions and density estimates of main foods. We found that body condition of parent owl males and females declined throughout the 40-year study period whereas the body condition of owlets at the fledging stage very slightly increased. The body condition of parent owls increased with augmenting depth of snow cover in late winter (January to March), and that of offspring improved with increasing precipitation in late spring (May to June). We conclude that the decreasing trend of body condition of parent owl males and females is important factor probably inducing reduced adult survival and reduced reproduction success thus contributing to the long-term decline of the Tengmalm’s owl study population. The very slightly increasing trend of body condition of offspring is obviously not able to compensate the overall decline of Tengmalm’s owl population, because the number of offspring in turn simultaneously decreased considerably in the long-term. The ongoing climate change appeared to work in opposite ways in this case because declining depth of snow cover will make the situation worse but increased precipitation will improve. We suggest that the main reasons for long-term decline of body condition of parent owls are interactive or additive effects of reduced food resources and increased overall predation risk due to habitat degradation (loss and fragmentation of mature and old-growth forests due to clear-felling) subsequently leading to decline of Tengmalm’s owl study population.

## Introduction

Physical condition of individuals is important for the ability to resist various parasites and diseases as well as in escaping predators thus contributing to reproductive success, over-winter survival and changes in wildlife population densities^1–3^. Although changes in physiology (e.g., body condition) as a trait level responses are relatively easy to measure in relation to changing climate worldwide^3,4^, it is surprising that they are less studied than phenological traits^5^. It was reported earlier that warmer temperatures have been connected with reduced body condition in both juvenile and adult birds^4,6, 7^. Responses in body condition to global warming are usual, but their direct influences on reproduction and then population growth seem to have only minor impacts regarding the total temperature on population dynamics^5^, which suggests that other unknown factors are probably involved. There also are evidences that weather conditions can directly influence densities of animal populations by alteration of reproductive success and/or survival, but the mechanisms of changes can also be indirect via modifying habitat and/or food abundance^8^. Recently, global changes in climatic conditions have been causing decreases in population densities of many different species, by worsening their reproductive success due, for instance, to phenological mismatches thus causing declines in population abundances which are in some causes leading up to local extinctions^9^.

Very little is known about the effects of long-term changes in weather conditions (climate-induced changes) on body condition of individuals on a population level from the long-term point of view, although food abundance in particular and also weather conditions are considered as key factors influencing the body condition, reproductive success, survival and population densities of birds and other animals^2,8^. Outside the breeding season, organisms cope with energetic challenges such as, for instance, disease, dispersion, challenging weather, and reduced food availability that can reduce energetic reserves available for future breeding, and these energetically demanding activities occurring during one life history stage can carry over to impact performance in a later stages^10–15^. Body condition of bird of prey adults and their offspring ultimately depends on food abundance and maintaining body condition may be related to age or the ability to defend good-quality territories^3^. Usually, yearling birds are in poorer condition during breeding season than older, more experienced individuals^16,17^. Females in good condition laid early and larger clutches and their condition decreased as the season advanced^18–21^. On the other hand, individuals in poor condition may not breed at all in a given year under conditions of food scarcity^22^. Body condition has also been used as a measure of health for a number of bird species^23–25^. Body condition of nestlings in asynchronously hatching bird species has been shown to be related to the hatching order within the nest and to the number of hatched individuals/brood size^23,26,27^.

Tengmalm’s owls (*Aegolius funereus*) are small predatory birds with almost exclusively nocturnal activity. They breed in cavities but readily accept also artificial nest boxes. The boreal zone coniferous forests and also alpine forests further south in the Holarctic region are their main habitats^28,29^. Tengmalm’s owls feed mainly on voles of the genera *Myodes* and *Microtus* (main prey) and during vole scarcity on shrews and small forest bird species (alternative prey)^30–33^. In this species, breeding duties are clearly defined. Males are food providers to females and their young during the whole nesting period and also during the post-fledging dependence period which may last 5 to 9 weeks^30,34–36^. The mean lifespan of Tengmalm’s owl males is 3.5 years on average and mean breading lifespan is about 1.5 years^32,37^. Female incubates the eggs, take care of the nestlings, and remains in the nest until the offspring are about 3 weeks old^30,38^. The eggs hatch in ca 2-day intervals (approximately according to the intervals in which the eggs are laid)^30^. For this reason, first-hatched young are on average 1 week older than last-hatched young^27^, which results in a marked size hierarchy among siblings. The duration of nestling period in individual young may take 27–38 days (from hatching to fledging), they leave the nest in the order as they hatched at intervals of about 1 day and the duration of fledging period increases with the number of siblings^38^.

The nation-wide population of Tengmalm’s owls in Finland was declining by 2% per year from the early 1980s to late 2010s resulting in overall decrease of population by about 70% up to 2019^32,39^. These population declines may be at least partly due to either decreased adult and/or juvenile body condition which subsequently induces reduced adult and/or juvenile survival, as well as reduced reproductive success. Recently, we showed that there was a decreasing long-term trend in the number of fledglings produced per breeding attempt from late 1970s to late 2010s, whereas there was no obvious decreasing trend in clutch size of Tengmalm’s owls^40^. Here we investigated long-term changes in physiology of the entire population, i.e., body condition of offspring at the fledging stage and parent male and female owls in relation to densities of main foods and weather variables during the period covering 34 years for offspring, 42 years for females and 40 years for males. This long-term data set offered us the unique opportunity to investigate whether: body condition of offspring at the fledging stage and parent male and female owls have decreasing long-term trends; interactive effects of abundance of main foods and weather conditions are at least partly inducing changes in body condition, and thus population declines. Following and in the line with our previous study^40^, in which we found long-term declining trends for breeding density and number of produced fledglings we predicted that (i) body condition of offspring and parent owls has decreasing long-term trends, too. Because clutches and broods are larger in good vole years than in poor ones^20,32^, (ii) offspring and parent owls would also be in better body condition in years with abundant main foods (voles) than in those of scarcity of main foods. Further, because older individuals surviving first year of their lives will have more experience and will be better hunters^32^, we predicted that (iii) body condition of parent owls increases with their age. Finally, because early breeders are usually the fittest and most productive individuals in the population^20,21,41,42^, we predicted that (iv) body condition of offspring decreases with delayed laying date, but (v) increases with body condition of parent owls in the extension of the third prediction^20,43,44^.

## Materials and methods

### Study area

We conducted the study in the Kauhava region of west-central Finland (approx. 63° N, 23° E). This lowland study area is only 30–120 m above sea level and around 61% of the study area is forested. Nearly all the forest areas are managed: first harvested by thinning when trees are 30–40 years old, and thereafter clear-cutting at intervals of 60–80 years. Nowadays old-growth forests comprise around 1% of the area. Clear-cut and sapling areas accounted for ca 7% of the study area, and agricultural land (mainly crop fields and pasture) covered 25%, peatland bogs 2%, other (settlements, roads etc.) 3%, and water (lakes, rivers, creeks) 2% of the area (for more details see^32,45^). Tengmalm’s owls breed in the natural cavities made by black woodpeckers (*Dryocopus martius*) in Europe but readily accept nest boxes. Nowadays, there are ca 450 nest boxes, which we inspected annually during 1973–2018, in the study area covering ca 1100 square km^32,45^.

We obtained meteorological data from the weather station located in the middle of the study area from the Finnish Meteorological Institute and included mean daily temperature (°C), total snow cover (cm), and daily precipitation (mm) during 1973–2018 (for details see^40^). Long-term trends for weather variables and their individual periods used in particular analyses are presented in the Supporting Information file (Supplementary S1 File).

### Field procedures

We visited wooden nest boxes in the study area in late March to late April and again in May to early June to find nests. The date of the laying of the first egg was obtained mainly by back-dating from hatching dates using 29 days as the incubation period for the first-laid egg^30^. We inspected the nests once per week (which is sufficiently often) until we found out the final number of eggs and hatchlings and to determine the hatching date (± 1 day). The age of the nestlings and their hatching order was based on the recorded date of hatching. All nestlings were ringed, and from 1985 onwards weighed and their wing length was measured at approximately the age of 25 days of the oldest owlet of the brood. We trapped a vast majority of parent females and males breeding in the study area during the middle of the nestling period. Parents were ringed, aged by checking the moult of primary and secondary feathers^32^, and we measured their wing length and body mass (females from 1977 and males from 1979 onwards; for trapping methods and measurement details, see^32^). Tengmalm’s owls can be reliably aged into three categories: 1-year, 2-year and older (3+) owls^32^.

The “scaled mass index” following the method by Peig and Green^46^ was calculated to quantify the body mass relative to the body size of owls. The regression slopes were 0.60 for offspring, 0.84 for males and 0.77 for females, whereas the average (± SD) wing lengths were 100.1 ± 19.83, 171.7 ± 3.69 and 178.9 ± 4.16 mm, respectively. Thus, we calculated the scaled mass index Ĥ_i_ (hereafter “BCI”, the body condition index) as follows: Ĥ_i_ = H_i_ (L_0_/L_i_)^bSMA^ where H_i_ and L_i_ are the body mass and the linear body measurement of individual i, respectively; b_SMA_ is the scaling exponent estimated by the standardised major axis (SMA) regression of H on L; L_0_ is an arbitrary value of L (e.g., the arithmetic mean value for the study population); and Ĥ_i_ is the predicted body mass for individual i when the linear body measure is standardized to L_0_ according to Peig and Green^46^.

We estimated abundances of main prey of Tengmalm’s owls (bank voles *Myodes glareolus*, field voles *Microtus agrestis* and sibling voles *Microtus rossiaemeridioinalis*) in the study area by snap-trapping each year in early May and in mid-September. Sampling was carried out in the four main habitat types (i.e., cultivated field, abandoned field, spruce forest, pine forest). Fifty-to-sixty baited Finnish metal mouse snap traps were set at 10 m intervals in vole runways on each sample plot and were checked daily for 3 consecutive days. The area of a sample plot was 0.5–0.6 ha, and the pooled trapping effort was approx. 600 trap-nights each year and season starting in 1973. The number of voles captured was standardized to the number of animals caught per 100 trap-nights (see^47^ for more details on trapping methods and vole cycles in the study area). As found earlier^47,48^, densities of bank and *Microtus* voles fluctuate in synchrony in the study area and the regional synchrony of vole population cycles extends up to 80 km, i.e., to the whole study area.

All field research protocols were approved by the Finish Museum of National History, Helsinki, Finland (ringing licence no. 524). The methods were carried out in accordance with the relevant guidelines and regulations of the Finish Museum of National History.

### Statistical analyses

A majority of the dataset used in this study was already published elsewhere^40^ when we analysed questions regarding the breeding performance of Tengmalm’s owls such as breeding densities, timing of breeding, clutch size and number of fledglings per breeding attempt. In the current study, we added body condition indices of parent owls and offspring in the dataset, and thus analysed the crucial second part of the whole long-term study.

We analysed the data with the aid of SAS System version 9.4 (SAS Institute Inc.) in three steps. The analysis checking for possible multicollinearity was done separately for the Tengmalm’s owl study population data (breeding density estimate, number of fledglings produced per breeding attempt, BCI of parent owl males and females, clutch size, laying date, male and female age), weather (mean of daily precipitation during May–June, mean of total snow cover during January–March), and prey abundance data (abundance of main prey in current spring and previous autumn). The two weather variables used in the three models (a–c, see below) were chosen based on the analyses and results of the above-mentioned study partly dealing with the same data set^40^. The two variables and their timespan (period) chosen fitted best our a priory hypotheses, were in line of the previous study, and other different periods were not tested compared to the previous analyses^40^. We first explored the correlations between the Tengmalm’s owl study population variables involved. Significant correlation was found between the number of eggs (E), hatchlings (H) and fledglings (F)—(EH: 0.86, P < 0.0001; EF: 0.44, P < 0.0001; HF: 0.54, P < 0.0001). We subsequently made a judgment of the extent of collinearity by checking related statistics, such as Tolerance value or Variance Inflation factor (VIF), Eigenvalue, and Condition Number following the approach of Schreiber-Gregory and Jackson^49^ and using TOL, VIF and COLLIN options of the MODEL statement in the SAS REG procedure. The lowest Tolerance value was 0.20949 and the highest VIF value was 4.77340. The relationship of the Eigenvalues (range 0.11965 to 1.59292) to the Condition Index values (range 4.54237 to 1.24493). So, there was no threat of multicollinearity indicated through these diagnostic results. Correlations are generally considered “strong” above 0.8^49^ or at least 0.5^50^. To avoid any possible interdependency, we still considered the number of eggs, hatchlings and fledgelings redundant so that only one of these three variables entered a statistical model.

We applied the same procedure for checking possible multicollinearity for mean of daily precipitation (mm) during May–June, mean of total snow cover (cm) during January–March, and abundance of main prey (voles) in current spring and previous autumn. There was only a high correlation between the abundance of main prey (voles) in the current spring or previous autumn (- 0.83, P < 0.0001). Therefore, only one of the two variables was used in further analyses. The lowest Tolerance value was 0.73587, and the highest VIF value was 1.35893. The relationship of the Eigenvalues (range 0.00357 to 0.22216) to the Condition Index values (range 32.10814 to 4.07254). Thus, neither in the weather and prey abundance data there was no indication of possible multicollinearity.

Second, using three different models (Table 1, a–c) analysed by Generalized Linear Fixed Model (GLM, PROC MIXED in SAS), associations were assessed between (a) body condition index of offspring, (b) body condition index of male parents, and (c) body condition index of female parents, and fixed and random effects. Fixed effects included or excluded according to alternative hypotheses within the three models (a–c) were: year (breeding season), breeding density estimate (number of nests/100 nest boxes), clutch size, number of fledglings produced per breeding attempt, laying date, hatching order, three categories of male and female age (1, 2 or 3+ years old), BCI of parent owl males and females (model a), mean of daily precipitation (mm) during May–June (model a), mean of total snow cover (cm) during January–March (models b and c), and abundance of main prey (voles) in current spring or previous autumn. Since we were mostly interested in the long-term changes of the dependant variables describing our study population, the fixed effect “year (breeding season)” was included in every single model (a–c). We performed all analyses using mixed model analysis with the individual nest, identity (ring number) of male and female parents as a random factor to account for the use of repeated measures on the same individuals. For the graphs, we estimated associations between the dependent variable and fixed effects (models a–c) by fitting a random coefficient model using PROC MIXED as described by Tao et al.^51^. We used non-temporal analytic framework for our statistical analysis instead of autocorrelation approach because mean breading lifespan of Tengmalm’s owls is about 1.5 years^37^, which does not allow meaningful autocorrelation analysis.

**Table 1:** Composition of the best models.

Model	AIC	Delta AIC	AIC weights wi	AIC odds	BIC	Delta BIC	BIC weights wi	BIC odds
**Model (a)—dependent variable body condition index of offspring**								
Year, spring prey abundance, male BCI, female BCI, laying date, No. of hatchlings, precipitation (May–June)	17,201.78	0.00	0.87	1.00	17,209.60	0.00	0.87	1.00
Year, spring prey abundance, male age, female age, laying date, No. of hatchlings, precipitation (May–June)	17,205.64	3.86	0.13	6.89	17,213.45	3.85	0.13	6.87
Year, spring prey abundance, male BCI, female BCI, laying date, No. of hatchlings	17,215.27	13.49	0.00	849.32	17,223.09	13.49	0.00	849.32
Year, spring prey abundance, male age, female age, laying date, No. of hatchlings	17,222.40	20.62	0.00	30,133.07	17,230.22	20.62	0.00	30,051.44
Year, spring prey abundance, male age, female BCI, laying date, No. of hatchlings, precipitation (May–June)	17,284.65	82.87	0.00	9.9E+17	17,292.48	82.88	0.00	9.94E+17
**Model (b)—dependent variable body condition index of male parents**								
Year, male age, spring prey abundance, snow cover (January–March)	6898.08	0.00	0.85	1.00	6910.33	0.00	0.85	1.00
Year, male age, autumn prey abundance, snow cover (January–March)	6902.18	4.10	0.11	7.79	6914.44	4.11	0.11	7.79
Year, male age, spring prey abundance	6904.46	6.38	0.04	24.34	6916.72	6.38	0.04	24.34
Year, male age, autumn prey abundance	6909.48	11.40	0.00	298.86	6921.73	11.40	0.00	298.86
Year, spring prey abundance, snow cover (January–March)	6947.28	49.20	0.00	4.8E+10	6959.53	49.20	0.00	4.82E+10
**Model (c)—dependent variable body condition index of female parents**								
Year, female age, autumn prey abundance, snow cover (January–March)	9113.99	0.00	0.78	1.00	9122.21	0.00	0.78	1.00
Year, female age, spring prey abundance, snow cover (January–March)	9116.50	2.50	0.22	3.49	9124.71	2.50	0.22	3.49
Year, female age, autumn prey abundance	9169.77	55.78	0.00	1.3E+12	9177.99	55.78	0.00	1.29E+12
Year, female age, spring prey abundance	9174.43	60.44	0.00	1.3E+13	9182.65	60.44	0.00	1.33E+13
Year, autumn prey abundance, snow cover (January–March)	9269.43	155.44	0.00	5.7E+33	9277.66	155.44	0.00	5.68E+33

Composition (applied fixed effects) of the five best fitting models sorted according to fitting statistics (the smaller the better), AIC, Δ AIC, and BIC, Δ BIC for all three modelled dependent variables (models a–c). The following fixed effects were log-transformed before the analyses: autumn and spring prey abundance, number of hatchlings and each weather variable.

Third, we applied model selection based on the information-theoretic paradigm using Akaike’s Information Criterion—IT-AIC^52^ and prepared a priori multiple hypotheses based on the remaining biologically relevant variables after testing for collinearity (each model/hypothesis tested including biological explanations of applied fixed effects are listed in the Supporting Information File—S1 File). There are warnings in the literature that Akaike Information Criterion (AIC)^53^ cannot be safely used in case of nested and mixed models^54,55^. Therefore, we used the two most important and frequent model selection criteria^55^, i.e., AIC, and Bayesian methods (BIC)^56^. Multiple information criteria are useful because each one was developed to optimize something different than the others. AIC is an example of efficient information criteria, while BIC is an example of consistent information criteria^57^. We found justification for such a procedure in a study of Posada and Buckley^58^. They showed that AIC and BIC are able to simultaneously compare multiple nested or non-nested models and assess model selection uncertainty^52^.

The differences (Δ_*i*_) between the Fit statistic values (the smallest values indicating the best fitting model) were sorted according to AIC values. Akaike weight *w*_*i*_ can be interpreted as the probability that M_*i*_ is the best model (in the AIC sense, that it minimizes the Kullback–Leibler discrepancy), given the data and the set of candidate models^59^. For five models with the lowest AIC values, we therefore calculated Δ AIC, Akaike weights *w*_*i*_, and for estimating the strength of evidence in favour of one model over the other we divided their Akaike weights *w*_*min*_*/w*_*j*_ (AIC Odds)^59^. As recommended by various authors^59–61^, using the same formulas just replacing AIC by BIC values, we obtained analogically Δ BIC, BIC weights *w*_*i*_, and BIC Odds. The advantage of this is that in comparison with AIC, BIC severely penalizes models with more parameters. Thus, the BIC weights *w*_*i*_ are appreciably different than for AIC weights *w*_*i*_^60^.

To find out whether the best model has merit, we compared our best model to the null model for all dependent variables using delta AIC (AIC null − AIC best model) and a relative information loss [exp((AIC_null − AICi_best)/2)], an approach adapted from Burnham and Anderson^59^. For each fixed effect in each model, we calculated coefficient estimates, standard errors, and 95% confidence intervals. The fixed effects could be considered significant when the 95% confidence interval did not include zero.

Finally, having the best composition of all models according to IT-AIC, these were calculated using GLMM, coefficient estimates, standard errors, and 95% confidence intervals were calculated for each model, and the results generated into the graphs.

## Results

In total, we recorded 1761 nests during 1973–2018 and handled (trapped, ringed or re-trapped, weighed, measured and aged) 1171 male parents during 1979–2018, 1468 female parents during 1977–2018, and 3971 offspring in 1044 broods during 1985–2018.

Table 1 shows five best fitting models sorted according to the fitting statistics (starting with the smallest value) for the three dependent variables tested (models a–c). In all cases Δ AIC, AIC weights *wi* and AIC Odds revealed comparable if not even identical results with Δ BIC, BIC weights *wi* and BIC Odds. This strengthened the credibility of the results. Comparison of the best model to the null model for all dependent variables is in Table 2 showing delta AIC (AIC null − AIC best model) and a relative information loss [exp((AIC_null − AICi_best)/2)].

**Table 2:** Comparison of the best models.

Comparing the best model to the null model	Dependent variable		
	Body condition index of offspring	Body condition index of male parents	Body condition index of female parents
Delta AIC (AIC null − AIC best model)	5837.04	116.4	246.2
Relative information loss [exp((AIC_null − AIC_best)/2)]	0	5.1417E−26	3.4356E−54

Comparison of the best model to the null model for body condition index of offspring, male and female parents (delta AIC and relative information loss).

### Body condition index (BCI) of offspring (a)

The composition of factors of the model with the lowest AIC and BIC values for the BCI of offspring had essential support with the probability of 87% that it is the best model (Table 1). The model with the second lowest AIC and BIC values had odds 6.89 times against it being the best model as compared to the best model in the candidate set. Therefore, the second and all the subsequent models did not need to be considered.

**Figure 1:**
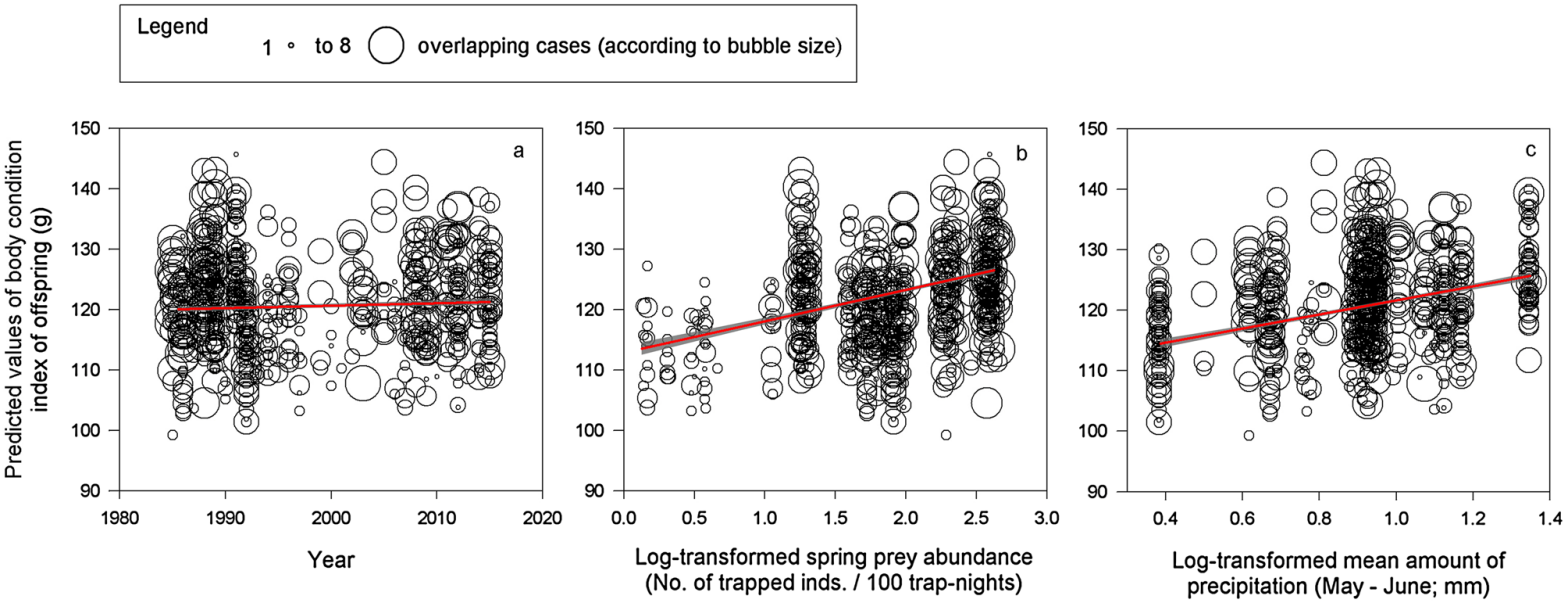
Body condition index of offspring. Bubble graph of predicted values of body condition index (BCI) of Tengmalm’s owl offspring during 1985–2018 plotted against year (**a**), log-transformed abundance index of main prey (voles) in the current spring (**b**), and log-transformed mean amount of precipitation during May to June (**c**) with regression line (red) and 95% confidence intervals (grey). The bubble size corresponds to the number of predicted (overlapping) cases which was between 1 and 8.

The best model explaining BCI of offspring (Table 2) included year, the log-transformed abundance of main prey in the current spring, BCI of parent males and females, laying date, log-transformed number of hatchlings, and log-transformed mean amount of precipitation during May to June (Table 1). Coefficient estimates, standard errors, and 95% confidence intervals are presented in Table 3. BCI of offspring slightly increased throughout the study period (1985–2018; Fig. 1a, Estimated slope [ES] = 0.05, Standard Error [SE] = 0.04, degrees of freedom [DF] = 1594), increased with log-transformed main prey abundance in the current spring (Fig. 1b, ES = 4.95, SE = 0.73, DF = 1752) and BCI of male and female parents (not shown, males: ES = 0.22, SE = 0.06, DF = 1639, females: ES = 0.15, SE = 0.03, DF = 1790). BCI of offspring further decreased with delayed laying date (not shown, ES =  − 0.14, SE = 0.03, DF = 1545) and number of hatchlings (not shown, ES =  − 9.73, SE = 2.24, DF = 1583). Finally, BCI of offspring augmented with log-transformed mean amount of precipitation during May to June (Fig. 1c, ES = 9.94, SE = 2.55, DF = 1643).

**Table 3:** Model information.

Model	Effect	β	SE	95% CI	
(a)—Body condition index of offspring	Intercept	− 28.08	89.90	− 204.41	148.26
	Year	0.05	0.04	− 0.04	0.14
	**Laying date**	− **0.14**	**0.03**	− **0.20**	− **0.09**
	**No. of hatchlings**	− **9.73**	**2.24**	− **14.13**	− **5.33**
	**Female BCI**	**0.15**	**0.03**	**0.10**	**0.20**
	**Male BCI**	**0.22**	**0.06**	**0.10**	**0.33**
	**Spring prey abundance**	**4.95**	**0.73**	**3.52**	**6.38**
	**Precipitation (May–June)**	**9.94**	**2.55**	**4.95**	**14.94**
(b)—Body condition index of male parents	**Intercept**	**242.22**	**50.68**	**142.72**	**341.72**
	**Year**	**− 0.07**	**0.03**	**− 0.12**	**− 0.02**
	Male age	− 0.14	0.27	− 0.66	0.39
	**Spring prey abundance**	**0.79**	**0.34**	**0.12**	**1.46**
	**Snow cover (January–March)**	**0.88**	**0.34**	**0.21**	**1.54**
(c)—Body condition index of female parents	**Intercept**	**944.97**	**89.99**	**768.42**	**1121.53**
	**Year**	**− 0.40**	**0.05**	**− 0.49**	**− 0.31**
	Female age	− 1.06	0.56	− 2.15	0.04
	Autumn prey abundance	− 0.41	0.73	− 1.84	1.03
	**Snow cover (January–March)**	**5.57**	**0.72**	**4.17**	**6.98**

Estimate (β), standard error (SE) and 95% confidence interval (CI) of the explanation variables in models with ΔAIC < 2 for the three models (Body condition index of offspring, male and female parents).

Variables with 95% CI that do not cross zero are shown in bold text. The following fixed effects were log-transformed before the analyses: autumn and spring prey abundance, number of hatchlings and each weather variable.

### Body condition index (BCI) of male parents (b)

The composition of factors of the model with the lowest AIC and BIC values for the BCI of male parents had essential support with the probability of 85% that it is the best model (Table 1). The model with the second lowest AIC and BIC values had odds 7.79 times against it being the best model as compared to the best model in the candidate set. Therefore, the second and all the subsequent models did not need to be considered.

**Figure 2:**
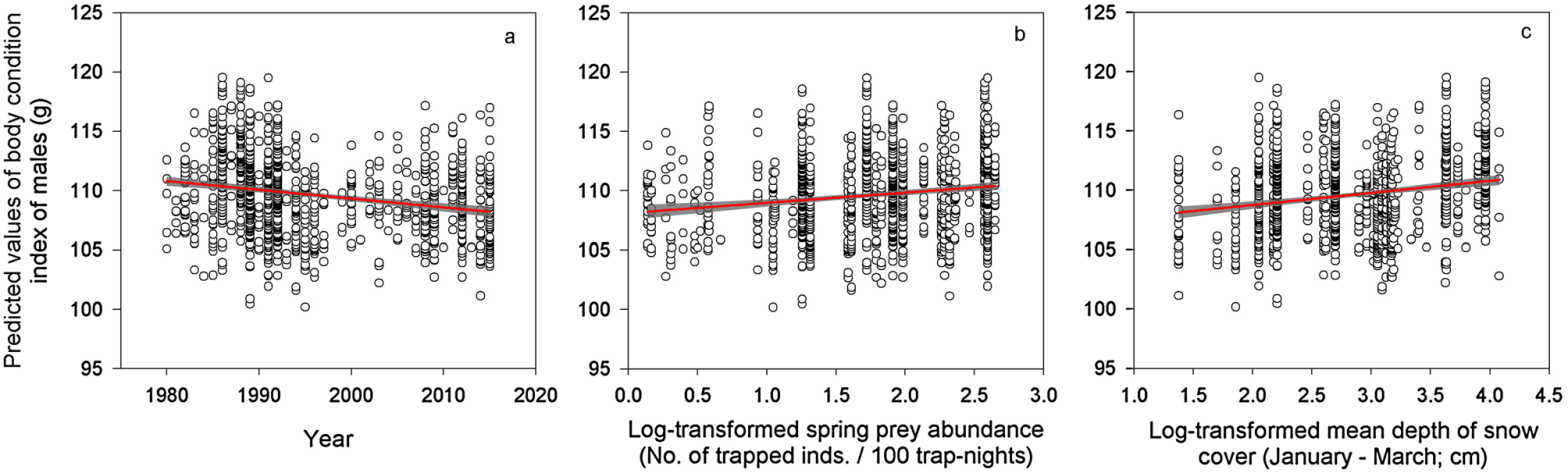
Body condition index of male parents. Predicted values of body condition index (BCI) of Tengmalm’s owl male parents during 1979–2018 plotted against year (**a**), log-transformed abundance index of main prey (voles) in the current spring (**b**), and log-transformed mean depth of snow cover during January to March (**c**) with regression line (red) and 95% confidence intervals (grey).

The best model explaining BCI of male parents (Table 2) included year, log-transformed main prey abundance in the current spring, male age, and log-transformed mean depth of snow cover during preceding January to March (Table 1). BCI of male parents declined throughout the study period (1979–2018; Fig. 2a, ES =  − 0.07, SE = 0.03, DF = 707), increased with log-transformed main prey abundance in the current spring (Fig. 2b, ES = 0.79, SE = 0.34, DF = 912), but slightly decreased with age (not shown, ES =  − 0.14, SE = 0.27, DF = 1001). Finally, BCI of male parents augmented with the log-transformed mean depth of snow cover during the previous January to March (Fig. 2c, ES = 0.88, SE = 0.34, DF = 976).

### Body condition index (BCI) of female parents (c)

The composition of factors of the model with the lowest AIC and BIC values for the BCI of female parents had essential support with the probability of 78% that it is the best model (Table 1). The model with the second lowest AIC and BIC values had odds 3.49 times against it being the best model as compared to the best model in the candidate set. Therefore, the second and all the subsequent models did not need to be considered.

**Figure 3:**
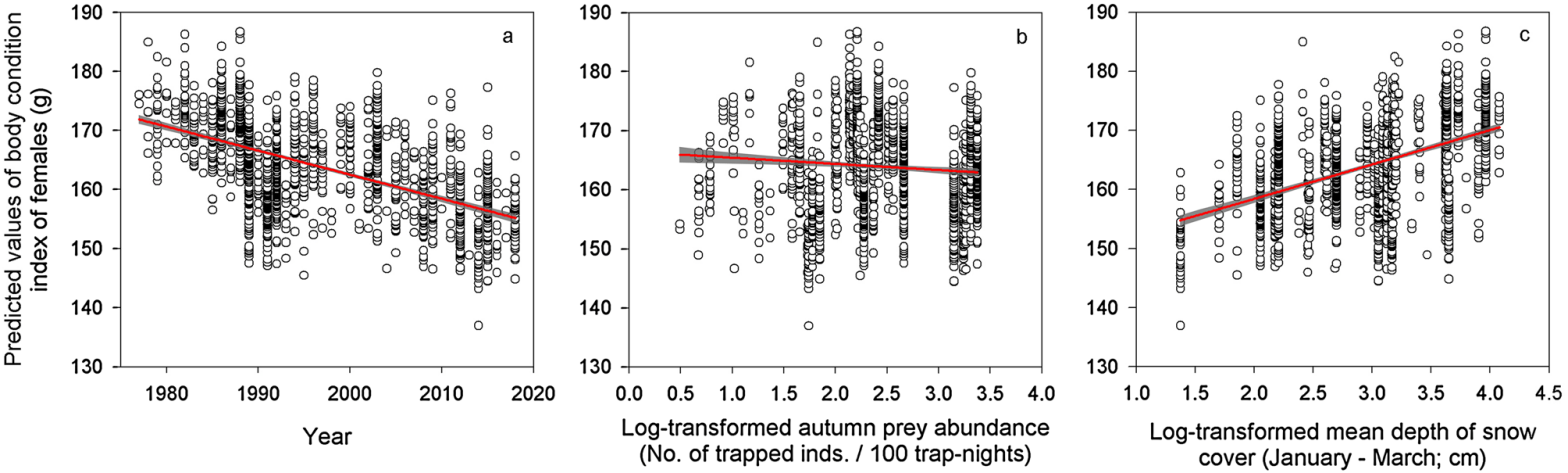
Body condition index of female parents. Predicted values of body condition index (BCI) of Tengmalm’s owl female parents during 1977–2018 plotted against year (**a**), log-transformed abundance index of main prey (voles) in the previous autumn (**b**), and log-transformed mean depth of snow cover during January to March (**c**) with regression line (red) and 95% confidence intervals (grey).

The best model explaining BCI of female parents (Table 2) included year, log-transformed main prey abundance in the previous autumn, female age, and log-transformed mean depth of snow cover during preceding January to March (Table 1). BCI of female parents declined throughout the study period (1977–2018; Fig. 3a, ES =  − 0.40, SE = 0.05, DF = 1235), decreased with log-transformed main prey abundance in the previous autumn (Fig. 3b, ES =  − 0.41, SE = 0.73, DF = 1333), and slightly decreased with age (not shown, ES =  − 1.06, SE = 0.56, DF = 1316). Finally, BCI of female parents augmented with the log-transformed mean depth of snow cover during the previous January to March (Fig. 3c, ES = 5.57, SE = 0.72, DF = 1312).

## Discussion

The main findings of this study were that body condition of both male and female parents of Tengmalm’s owls showed declining long-term trend, whereas the body condition of their offspring slightly increased from 1980 to 2010s. In addition, depth of snow cover emerged as the most important weather variable modifying the body condition of parent males and females, as well as the amount of precipitation appeared as the most important factor affecting the body condition of offspring in the long-term.

Contrary to our expectations, body condition of offspring was very slightly increasing (or remained practically stable) throughout the study period. This result might be explained by the fact that the fledgling production per breeding attempt by the same parent owls decreased during the same time period^40^. The main reason for marked decline in offspring production was brood reduction due to lack of food inducing starvation of owlets during the nestling period. Valkama et al.^27^ showed that the proportion of hatchlings producing fledglings decreased with the degree of asynchrony within broods during the decrease phase of the vole cycle, which showed that nestling mortality was most frequent among asynchronously-hatched broods when food became scarce. Therefore, when owls raised overall smaller broods these offspring could consequently be in slightly better condition. This result is consistent with Lack’s^62^ brood reduction hypothesis stating that if food becomes scarce during the nestling period the youngest nestlings would die first without endangering the survival of the whole brood. This further corresponds well with the result that body condition of offspring decreased with increasing number of hatchlings, as it was also found, for instance, in burrowing owls (*Athene cunicularia*)^26^.

The finding that body condition of offspring increased with increasing amount of precipitation during the current spring (May to June) is consistent with our previous result that the number of fledglings produced per breeding attempt also augmented with the amount of precipitation during the same time period^40^. It was earlier found that increased summer temperatures improved vole densities and maintained 3-year high-amplitude cycles of vole populations in South and Central Finland^63^. Warm and humid spring and summer seasons have positive effects on vole densities via improved food supply of herbivorous voles, and thus increase both body condition and offspring production of Tengmalm’s owls^40^.

As expected, the body condition of offspring amplified with augmenting abundance of main foods in the current spring. This result reveals the importance of limitation in main food resources during the nestling period for body condition of offspring, and thus for their future survival. This interpretation is also supported by the results from supplementary feeding experiments in both Tengmalm’s owls and Eurasian kestrels (*Falco tinnunculus*) from hatching onwards. Offspring of both species were in better body condition in supplementary fed nests than in non-supplemented control nests^25,64^. These results are consistent with results on recruitment rate of Tengmalm’s owl offspring to the future breeding population, which is two-to-three times higher for fledglings hatched in the increasing phase of the vole cycle with improving conditions of main foods than in the decrease and low phases of the vole cycle with deteriorating or poor abundances of main foods^65^. Similarly, in passerine birds better food availability during the nestling period improved growth, development, body condition and survival of nestlings, and can also positively impact post-fledging survival and recruitment probability^66–72^, but contradictory findings were also reported^73–75^. In a similar way, better body condition, post-fledging survival and recruitment probability thanks to higher food availability was also recorded in several bird of prey species^76–80^, but not in Ural owls (*Strix uralensis*) subsisting mainly on voles^1^. Thus body condition at fledging obviously have carryover effects in post-fledging survival which in turn is crucial for reproductive success with ultimate consequences for individual fitness and population dynamics^77,81,82^.

The body condition of offspring also increased with body condition of male and female parents and decreased with later laying date. These results are consistent with findings that adult birds of prey in better body condition are usually the earliest breeders^17,20,41,42,83^, and that more experienced adults breed earlier in the season^32,40,44^. Positive correlation between body condition of offspring and their parents was also found in passerine species^84^.

Body condition of parent owl males and females showed declining long-term trend during late 1970s to late 2010s. The long-term decline of body condition was more pronounced in females than male owls, thus making this issue even more serious from the conservation point of view, because body condition of females is decisive for breeding success in Tengmalm’s owls^16,32,85^. Moreover, earlier studies showed that the physiological state of birds including body condition contributes to its reproductive success and survival^3,15,18,86–88^. These results correspond well to the declining long-term trends in breeding densities and fledgling production per breeding attempt as found previously in our study area^40^.

Contrary to our expectations, the body condition of parent owls was slightly decreasing with their age. We can speculate the older and more experienced individuals do not need to make body reserves as large as yearlings because they are better able to capture some prey on the daily basis. This can be supported by the fact that adults had greater hunting success than juveniles in nine raptor species^89^. In a similar way, it was shown that hatch-year pygmy owls (*Glaucidium passerinum*) hoarded more food items in their winter food stores and during low vole years stored less birds (alternative prey much harder to catch) than the adults^90,91^. This suggests that less-experienced yearlings rely more on stored food than adults^90^, and thus supports the explanation that experienced individuals are better able to capture some prey on the daily basis as mentioned above. Alternatively, this result might arise from the fact that yearling owls, particularly yearling males, usually breed only in good vole years^32^, thus being in better body condition compared to older individuals breeding also during declining and/or low phase of the vole cycle and being most probably in worse body condition at that time. Further, we should keep in mind that the parent owls were trapped and weighed during the mid-nestling period in spring. It is simply possible that we could find the opposite (older owls to be heavier than yearlings) in the middle of winter (when it is better to have more fat reserves) as found, for instance, in snowy owls (*Bubo scandiacus*)^3^.

We further found that body condition of parent owls increased with increasing height of snow cover during the preceding winter (January to March) similarly as we previously found for clutch size of Tengmalm’s owls^40^. We suggest that deep snow cover in the course of winter offers effective insulation for over-wintering voles that can even reproduce below the deep snow cover^92^, where the ambient temperature is relatively constantly close to 0 °C. Deep snow cover may thus have a positive effect on overwinter survival of voles^93–95^, and may result in higher vole densities in early spring, which in turn may induce larger clutch sizes and better body condition of parent owls.

We conclude that the decreasing trend of body condition of parent owl males and females is important factor probably inducing reduced adult survival, as well as reduced reproduction success thus contributing to the long-term decline of the Tengmalm’s owl study population. The very slightly increasing trend of body condition of offspring is obviously not able to compensate the overall decline of Tengmalm’s owl population, because the number of fledglings in turn simultaneously decreased considerably in the long-term^40^. The ongoing climate change appeared to work in opposite ways in this case because declining depth of snow cover will make the situation worse (in terms of lowered over-winter survival and densities of voles in early spring, and thus smaller clutch sizes and worse body condition of parent owls), whereas increased precipitation will improve the situation (in terms of higher densities of herbivorous voles via improved food supply, and thus increased offspring body condition and fledgling production). We suggest that the main reasons for long-term decline of body condition of parent owls are probably interactive or additive effects of reduced food resources and increased predation risk due to habitat degradation: loss and fragmentation of mature and old-growth forests due to clear-felling. Degradation of forest habitat subsequently leads to decline of local Tengmalm’s owl population^40^ and nation-wide population in Finland^32,39,96^. It seems that Tengmalm’s owls are not able to cope with multiple stressors induced by forest habitat degradation which have negative carry-over effects in the long-term regarding particularly parents’ body condition and breeding success, and most probably also over-winter survival. In addition, the situation is also quite similar and serious for many other forest-dwelling specialists inhabiting boreal forests^97–104^, and it will only deteriorate over time unless major measures to reverse the degradation of forest habitat due to clear-felling are taken and actually implemented as soon as possible.

## Supplementary Information

Supplementary Information.
